# The striking geographical pattern of gastric cancer mortality in Spain: environmental hypotheses revisited

**DOI:** 10.1186/1471-2407-9-316

**Published:** 2009-09-08

**Authors:** Nuria Aragonés, Beatriz Pérez-Gómez, Marina Pollán, Rebeca Ramis, Enrique Vidal, Virginia Lope, Javier García-Pérez, Elena Boldo, Gonzalo López-Abente

**Affiliations:** 1 Environmental and Cancer Epidemiology Unit, National Center for Epidemiology, Carlos III Institute of Health, Madrid, Spain; 2 Consortium for Biomedical Research in Epidemiology & Public Health (CIBER en Epidemiología y Salud Pública - CIBERESP), Parc de Recerca Biomèdica de Barcelona, Doctor Aiguader, 88 1ª Planta, 8003 Barcelona, Spain

## Abstract

**Background:**

Gastric cancer is decreasing in most countries. While socioeconomic development is the main factor to which this decline has been attributed, enormous differences among countries and within regions are still observed, with the main contributing factors remaining elusive. This study describes the geographic distribution of gastric cancer mortality at a municipal level in Spain, from 1994-2003.

**Methods:**

Smoothed relative risks of stomach cancer mortality were obtained, using the Besag-York-Molliè autoregressive spatial model. Maps depicting relative risk (RR) estimates and posterior probabilities of RR being greater than 1 were plotted.

**Results:**

From 1994-2003, 62184 gastric cancer deaths were registered in Spain (7 percent of all deaths due to malignant tumors). The geographic pattern was similar for both sexes. RRs displayed a south-north and coast-inland gradient, with lower risks being observed in Andalusia, the Mediterranean coastline, the Balearic and Canary Islands and the Cantabrian seaboard. The highest risk was concentrated along the west coast of Galicia, broad areas of the Castile & Leon Autonomous community, the province of Cáceres in Extremadura, Lleida and other areas of Catalonia.

**Conclusion:**

In Spain, risk of gastric cancer mortality displays a striking geographic distribution. With some differences, this persistent and unique pattern is similar across the sexes, suggesting the implication of environmental exposures from sources, such as diet or ground water, which could affect both sexes and delimited geographic areas. Also, the higher sex-ratios found in some areas with high risk of smoking-related cancer mortality in males support the role of tobacco in gastric cancer etiology.

## Background

Gastric cancer has plotted a trend very different to that of other malignant tumors in recent decades, with a marked decline in incidence and mortality, described by the scientific community as an "unplanned triumph" [1]. However, this tumor still ranks fourth in terms of incidence and second in cancer mortality worldwide [2]; in 2002, there were more than 900,000 new cases of gastric cancer around the world, 66% of which occurred in less developed countries [3].

One of this tumor's epidemiologic characteristics is the presence of marked geographic differences worldwide. The highest incidence rates have been reported in Korean and Japanese cancer registries, where rates are tenfold those of the United States. Interestingly, the world geographic risk pattern is very similar in both sexes, with the sex ratio being stable -generally in the order of 2- across high and low incidence regions [3]. This fact suggests that environmental exposures might play an important role in this tumor's carcinogenesis, which is not yet well understood.

Overall, gastric tumors account for more than 90% of adenocarcinomas (AC) but there are two well-differentiated AC groups, namely, intestinal and diffuse type [4], with different clinical, epidemiologic and pathologic characteristics. The intestinal type is more prevalent among men and the elderly, tends to be sited in the noncardia portions of the stomach, and is predominant in the lowest socioeconomic groups and high risk areas. Furthermore, it is the type to which the decline in gastric cancer in high-risk populations has been attributed [5,6]. Diet and H. pylori infection are considered the most important factors involved in this type of cancer. Diffuse AC, with an M:F ratio bordering on unity, is the most usual histological type in gastric cardia neoplasms, is more frequent among the young, and has been linked to constitutionally-related factors [7,8].

As mentioned above, gastric cancer has been related with socioeconomic status. At an individual level, this variable can be linked to dietary patterns, infection by Helicobacter pylori, tobacco use, and, to a lesser extent, occupational exposures taking place in less qualified jobs [9,10]. At an ecologic level, this variable might reflect differences in environmental exposures associated with pollution and other hazardous exposures [11,12]. Yet, the relationship between this variable and gastric cancer frequency is not universally robust, since countries with a high socioeconomic level, such as Japan, maintain high rates of this disease.

In Spain, previous geographic studies using provinces as study units have shown that gastric cancer mortality rates displayed a singular spatial distribution, which was similar across the sexes and different from that of any other tumor [13]. Moreover, this pattern -with some changes- has been very consistent over recent decades. In order to improve the description of the high-risk areas, in this paper we use data aggregated at municipal level, the smallest geographic administrative boundaries that can be used for the whole country. This approach presents some limitations, since sparsely populated areas with few or zero cases can generate extreme RR values. However, recent advances in the field of spatial epidemiology have opened the way to new methods of disease mapping which enable these challenges to be successfully met [14]. The most widely used strategy for tackling the problems posed by small-area analysis is to estimate the spatial distribution of risk by means of simulation based on Bayesian hierarchical models. Analysis of small areas improves the interpretation of results and the capacity to detect local effects linked to environmental problems, while reducing ecologic biases.

The objective of this study was to show the spatial distribution patterns of gastric cancer mortality in men and women in Spain, and help to generate new hypotheses which might serve to explain these patterns. On the assumption that lung cancer mortality is linked to the prevalence of tobacco smokers, we also show maps depicting the municipal distribution of lung cancer mortality in Spain.

## Methods

As case source, we used individual death entries for the period 1994-2003 corresponding to gastric cancer (International Classification of Diseases, 9^th ^revision [ICD-9], code 151) and lung cancer (ICD-9 code 162). These data, which include information on town of residence at death, were supplied by the National Statistics Institute broken down by age group (18 groups) and sex. The municipal populations, also broken down by age group and sex, were drawn from the 1996 electoral roll and 2001 census. These years correspond to the midway points of the two quinquennia that comprise the study period (1994-1998 and 1999-2003). The person-years for each five-year period were estimated by multiplying these populations by 5.

The methodology has been explained elsewhere in more detail [15]. Briefly, gastric cancer standardized mortality ratios (SMRs) were computed as the ratio between the observed and the expected number of deaths. For the calculation of expected cases, the overall age-specific Spanish mortality rates for the two 5-year periods were multiplied by each town's person-years, broken down by age group, sex, and quinquennium.

Smoothed municipal relative risks (RRs) for map-plotting purposes were estimated, by fitting spatial Poisson models with two random-effects terms that took the following into account: a) municipal contiguity (spatial term); and b) municipal heterogeneity. These models come within the category of the so-called conditional autoregressive (CAR) models proposed by Besag, York and Mollié [16], and were fitted using Bayesian Markov chain Monte Carlo simulation methods with non-informative priors [17]. Convergence of the simulations was verified using the BOA (Bayesian Output Analysis) R program library [18]. Given the great number of parameters of the models, the convergence analysis was performed on a randomly selected sample of 10 towns and cities, taking strata defined by municipal size. Posterior distributions of relative risk were obtained using WinBugs [19]. The criterion of contiguity used was adjacency of municipal boundaries. Results from these models were included in a Geographic Information System to plot maps that depicted smoothed RR estimates and the distribution of the posterior probability that RR>1 (Bayesian version of p value). Insofar as this indicator is concerned, probabilities above 0.8 should be deemed statistically significant [20]. Thereafter, we calculated the ratio of estimated RRs in males and females by municipality.

Additionally, a similar model was constructed for lung cancer (only men); its spatial pattern might be considered a surrogate indicator of the smokers male prevalence in Spain.

## Results

From 1994 to 2003, a total of 62184 gastric cancer deaths were registered in Spain (37963 in men, and 24221 in women), accounting for 7% of all deaths due to malignant tumors nationwide in this period. Table 1 lists a number of descriptive statistics for both sexes.

**Table 1:** Summary of population and gastric cancer mortality in Spain's 8072 towns and cities, 1994-2003.

	Total	Mean	Standard Deviation	**Min**.	**Max**.	P10	Median	P90	No. (%) with zero counts
**MEN**									
Population	19698855	2440	20161	3	1356000	48	293	3626	0
Observed	37963	4.70	39.36	0	2746	0	1	7	3205
Expected	38199	4.73	41.66	0.01	2874	0.19	0.94	7.24	0
SMR	-	1.04	1.61	0	28.57	0	0.69	2.59	3205
RR	-	1.08	0.22	0.41	2.55	0.83	1.04	1.38	0
**WOMEN**									
Population	20549210	2545	22612	1	1547000	43	281	3690	0
Observed	24221	3.00	28.01	0	1959	0	0	5	4156
Expected	24315	3.01	29.97	0.00	2089	0.10	0.54	4.50	0
SMR	-	1.07	2.28	0	50.00	0	0	2.75	4156
RR	-	1.10	0.25	0.23	2.63	0.84	1.06	1.41	0

**Figure 1:**
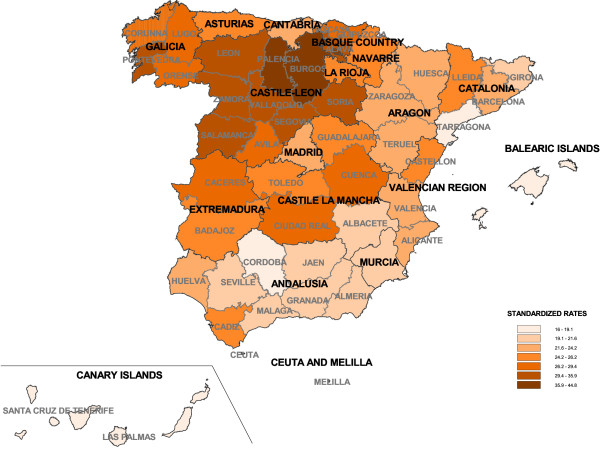
**Provincial age-standardized gastric cancer mortality rates (both sexes)**. Spain, 1994-2003.

To give an overall picture, Figure 1 shows age-standardized rates of gastric cancer mortality by province. Also by way of reference, Table 2 presents the provincial age-standardized rates (ASR) of gastric cancer mortality by sex. The province with highest mortality in both sexes was Burgos (ASR in men: 31.01; ASR in women: 13.81), followed by Palencia y Pontevedra. In contrast, Santa Cruz de Tenerife and the Balearic Islands presented the lowest mortality rates in men and women respectively (ASR in men 10.88 and ASR in women 4.46).

**Table 2:** Age-standardized gastric cancer mortality rates by sex and province in Spain, 1994-2003.

		Men		Women		
				
Autonomous Region	Province	Observed cases	ASR	Observed cases	ASR	Male:female Ratio
Andalusia	Almería	331	14.14	193	6.41	2.21
	Cádiz	828	18.35	485	7.51	2.44
	Córdoba	554	13.67	316	5.44	2.51
	Granada	620	14.35	399	6.87	2.09
	Huelva	364	15.75	243	7.56	2.08
	Jaén	527	14.59	302	6.80	2.15
	Málaga	843	14.33	425	5.20	2.76
	Sevilla	1126	14.93	650	5.83	2.56
Aragon	Huesca	284	15.77	192	8.43	1.87
	Teruel	178	15.00	126	8.14	1.84
	Zaragoza	875	15.60	631	7.47	2.09
Asturias	Asturias	1242	17.45	875	7.83	2.23
Balearic Islands	Balearic Islands	485	11.61	266	4.46	2.60
Basque Country	Alava	351	22.87	198	9.37	2.44
	Guipuzcoa	748	19.74	410	7.25	2.72
	Vizcaya	1312	20.33	726	7.55	2.69
Canary Islands	Las Palmas	440	12.70	250	5.40	2.35
	St.Cruz Tenerife	397	10.88	254	5.11	2.13
Cantabria	Cantabria	544	16.59	333	6.49	2.56
Castile la Mancha	Albacete	308	14.64	189	6.95	2.11
	Ciudad Real	560	19.35	349	8.20	2.36
	Cuenca	310	19.22	161	7.77	2.47
	Guadalajara	214	17.81	128	8.42	2.12
	Toledo	601	17.95	382	8.18	2.20
Castile-Leon	Avila	315	21.30	160	8.09	2.63
	Burgos	740	31.01	479	13.81	2.25
	Leon	813	21.90	513	9.35	2.34
	Palencia	355	28.38	244	12.64	2.25
	Salamanca	561	21.31	357	9.86	2.16
	Segovia	275	24.05	169	10.71	2.25
	Soria	191	22.24	126	10.70	2.08
	Valladolid	657	22.74	415	9.69	2.35
	Zamora	385	21.90	244	10.08	2.17
Catalonia	Barcelona	4063	15.41	2679	6.65	2.32
	Girona	465	13.89	330	6.87	2.02
	Lleida	474	17.35	276	7.44	2.33
	Tarragona	475	13.13	277	5.66	2.32
Extremadura	Badajoz	666	17.90	360	6.75	2.65
	Cáceres	536	19.98	349	8.82	2.27
Galicia	A Coruña	1317	19.84	907	8.71	2.28
	Lugo	567	17.73	403	9.14	1.94
	Ourense	518	17.50	416	9.24	1.89
	Pontevedra	1144	24.21	906	11.69	2.07
La Rioja	La Rioja	323	18.10	180	7.51	2.41
Madrid	Madrid	4084	15.92	2799	7.00	2.27
Murcia	Murcia	781	14.29	540	7.29	1.96
Navarre	Navarre	599	17.93	376	7.83	2.29
Valencian Region	Alacant	1165	15.55	687	7.02	2.22
	Castello	483	16.76	307	8.11	2.07
	Valencia	1895	16.23	1187	7.05	2.30
Ceuta	Ceuta	46	16.18	28	6.85	2.36
Melilla	Melilla	28	10.01	25	7.71	1.30

ASR = Age-Standardized Rate.

**Figure 2:**
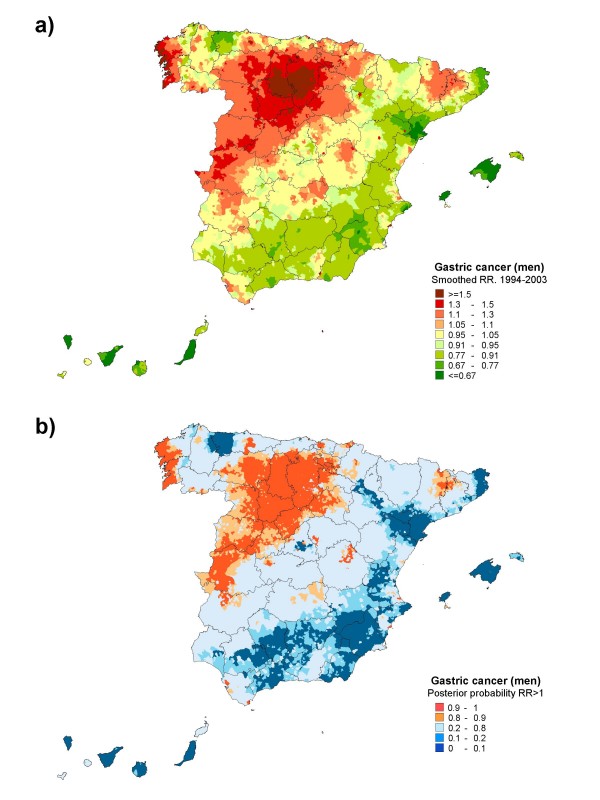
**Municipal distribution of gastric cancer mortality in men: a) smoothed relative risk (RR); b) posterior probability of RR being greater than 1**. Spain, 1994-2003.

**Figure 3:**
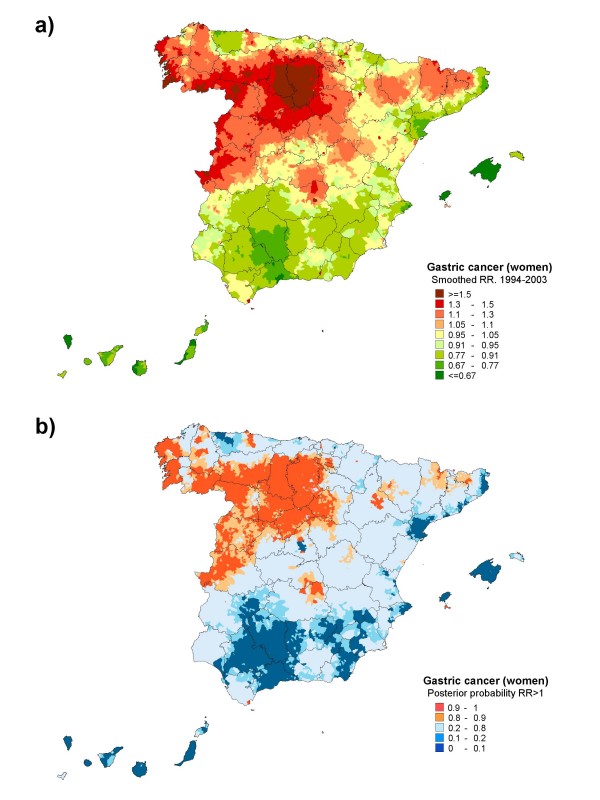
**Municipal distribution of gastric cancer mortality in women: a) smoothed relative risk (RR); b) posterior probability of RR being greater than 1**. Spain, 1994-2003.

Figures 2 and 3 depict the smoothed RRs for males and females, together with the spatial distribution of posterior probabilities of having a relative risk greater than 1 in each sex. The smoothed RR maps enable homogeneous areas to be delimited. In men, there was a huge area of excess risk that covered: the Autonomous Community of Castile & Leon; the western half of Cáceres and the north of Badajoz in Extremadura; specific areas in the provinces of Toledo, Guadalajara, Cuenca and Ciudad Real in Castile-La Mancha; La Rioja; and, Navarre and the Basque Country. In addition, there were two areas with clear excess risk, i.e., the Atlantic coast of Galicia and the interior of Catalonia, comprising the districts of Ripollés in the province of Girona, and Pallars Sobirá and Alto Urgel in Lleida.

While the general pattern was very similar among women, some differences were nevertheless in evidence. The excess risk area corresponding to Castile & Leon was larger, in that it included almost the entire province of Cáceres, all four provinces of Galicia, and most of the territory in Aragon. In contrast, no increased risk was observed in the northern areas of the Basque Country and Navarre. A noteworthy feature was the marked south-north and coast-inland pattern of gastric cancer mortality in both sexes, with a relatively significant, low risk of dying from this cancer in the Spain's Andalusian and Mediterranean provinces, the Canary and Balearic Islands, and part of the Cantabrian coastline.

Shown in Table 3 are the SMRs and RRs for a selection of towns with excess risk of gastric cancer mortality. Towns were required to have RRs of over 1.50, based on a difference between observed and expected numbers equal to or greater than 3 cases, and a posterior probability of over 0.9: a total of 67 towns, belonging to 7 Autonomous Communities, met these criteria. A total of 55% of the towns selected were situated in Galicia, specifically in the provinces of Pontevedra and Corunna. Attention should be drawn to the fact that 9 of the 10 towns with highest excess risk in men and women were Galician, and that 7 of these, all lying in the province of Pontevedra, were the same for both sexes (Bueu, Cangas, A Guarda, Vilaboa, Moaña, O Grove and Marín). Indeed, Bueu and Cangas were the towns that registered the highest RRs in the whole of Spain, for men and women alike.

**Table 3:** Towns with 5 or more gastric cancer deaths which have shown RRs of over 1.5 in men or women, based on a difference between the number of observed and expected deaths equal to or greater than 3, and a posterior probability > = 0.9 (1994-2003).

Autonomous region	Province	Town	Males				Females			
			Observed	Expected	SMR	RR	Observed	Expected	SMR	RR
Asturias	Asturias	Langreo	93	52.1	1.79	**1.57**	45	36.7	1.23	**1.21**
Castile La Mancha	Ciudad Real	Solana (la)	21	14.1	1.49	**1.18**	22	8.4	2.63	**1.51**
Castile & Leon	Burgos	Aranda de Duero	38	26.4	1.44	**1.45**	31	15.6	1.99	**1.68**
		Belorado	7	3.3	2.12	**1.48**	7	2.0	3.44	**1.55**
		Briviesca	13	6.2	2.10	**1.52**	8	4.0	2.02	**1.58**
		Burgos	341	151.4	2.25	**2.13**	208	102.3	2.03	**1.97**
		Ibeas de Juarros	5	1.5	3.29	**1.74**	1	0.7	1.40	**1.75**
		Lerma	5	2.9	1.74	**1.63**	5	1.8	2.71	**1.94**
		Salas de los Infantes	7	2.9	2.38	**1.63**	1	1.6	0.63	**1.49**
		Sasamón	8	2.7	2.94	**1.62**	2	1.5	1.34	**1.73**
	Leon	Bustillo del Páramo	4	3.1	1.29	**1.30**	7	1.7	4.20	**1.59**
		Santa María del Páramo	3	3.2	0.93	**1.33**	8	1.9	4.12	**1.76**
		Villarejo de Órbigo	1	5.2	0.19	**1.15**	9	3.1	2.88	**1.52**
	Palencia	Becerril de Campos	6	1.8	3.35	**1.63**	4	1.3	3.19	**1.63**
		Carrión de los Condes	6	3.0	2.00	**1.58**	4	2.6	1.56	**1.54**
		Dueñas	7	3.4	2.04	**1.59**	2	1.8	1.12	**1.57**
		Palencia	133	76.7	1.74	**1.68**	89	53.2	1.67	**1.65**
		Saldaña	12	3.6	3.30	**1.59**	7	2.3	3.06	**1.55**
		Venta de Baños	9	6.4	1.40	**1.55**	8	4.1	1.96	**1.69**
		Villada	4	1.9	2.13	**1.51**	7	1.6	4.41	**1.52**
		Villarramiel	5	1.4	3.62	**1.58**	1	1.1	0.89	**1.42**
	Salamanca	Bejar	39	19.8	1.97	**1.57**	22	13.6	1.62	**1.34**
		Hinojosa de Duero	2	1.6	1.24	**1.20**	6	1.1	5.59	**1.53**
	Valladolid	Laguna de Duero	13	7.4	1.75	**1.51**	5	3.8	1.32	**1.37**
		Peñafiel	10	5.9	1.69	**1.48**	10	3.8	2.60	**1.67**
	Zamora	Gallegos del Río	2	1.8	1.14	**1.19**	5	1.0	5.12	**1.60**
Valencian Region	Alicante	Santa Pola	35	15.4	2.27	**1.84**	8	8.6	0.93	**0.99**
Cantabria	Cantabria	Reinosa	22	13.4	1.64	**1.51**	7	8.9	0.79	**0.97**
Extremadura	Badajoz	Calamonte	7	4.7	1.48	**1.28**	12	2.7	4.39	**2.00**
	Cáceres	Membrio	1	1.7	0.59	**1.25**	6	1.1	5.38	**1.53**
Galicia	Corunna	Boiro	31	16.0	1.94	**1.75**	15	11.0	1.37	**1.41**
		Camariñas	20	6.7	2.96	**2.02**	6	4.5	1.33	**1.38**
		Carnota	16	7.2	2.24	**1.82**	10	5.6	1.78	**1.51**
		Cee	12	7.0	1.72	**1.51**	7	5.0	1.41	**1.41**
		Lousame	14	5.7	2.48	**1.69**	5	4.0	1.24	**1.31**
		Malpica de Bergantiños	13	8.5	1.53	**1.32**	13	5.8	2.24	**1.63**
		Mazaricos	15	7.1	2.12	**1.57**	6	5.7	1.06	**1.33**
		Muros	28	10.5	2.66	**2.00**	16	8.2	1.95	**1.55**
		Noia	26	12.8	2.04	**1.73**	15	9.9	1.51	**1.38**
		Porto do son	17	10.1	1.69	**1.70**	14	7.7	1.81	**1.55**
		Puebla del Caramiñal	17	9.3	1.82	**1.73**	12	6.8	1.77	**1.61**
		Ribeira	37	23.2	1.60	**1.64**	28	15.4	1.81	**1.71**
	Lugo	Xove	2	4.5	0.45	**1.02**	8	2.8	2.91	**1.58**
	Orense	Cualedro	5	4.9	1.02	**1.09**	10	2.7	3.66	**1.64**
		Monterrei	5	6.9	0.72	**1.10**	8	3.9	2.06	**1.67**
		Verín	28	14.7	1.90	**1.38**	25	9.0	2.77	**1.95**
	Pontevedra	Bueu	33	10.4	3.18	**2.55**	21	6.8	3.08	**2.63**
		Cambados	18	10.8	1.66	**1.61**	9	7.1	1.26	**1.63**
		Cangas	49	19.0	2.58	**2.46**	31	12.7	2.44	**2.48**
		Gondomar	15	9.1	1.65	**1.40**	11	5.9	1.85	**1.57**
		Grove (o)	21	9.6	2.19	**1.89**	18	6.3	2.84	**2.33**
		Guarda (a)	25	8.5	2.94	**2.43**	16	6.1	2.62	**2.36**
		Marín	35	19.1	1.83	**1.87**	27	13.2	2.05	**2.10**
		Meis	9	4.9	1.83	**1.52**	7	3.8	1.83	**1.54**
		Moaña	23	14.3	1.61	**1.95**	27	9.7	2.77	**2.50**
		Mos	16	11.2	1.43	**1.40**	12	7.5	1.59	**1.54**
		Neves (as)	9	6.0	1.51	**1.29**	10	4.4	2.26	**1.65**
		Nigrán	11	11.8	0.93	**1.24**	13	8.3	1.57	**1.54**
		Ponteareas	24	15.9	1.51	**1.38**	27	11.1	2.44	**1.74**
		Redondela	39	22.9	1.70	**1.52**	22	16.0	1.38	**1.45**
		Rosal (o)	8	6.1	1.30	**1.66**	11	4.4	2.52	**2.00**
		Salvaterra do Miño	14	8.6	1.64	**1.35**	15	6.5	2.31	**1.66**
		Sanxenxo	18	14.5	1.24	**1.43**	14	9.3	1.51	**1.64**
		Tui	20	13.9	1.44	**1.39**	16	10.7	1.49	**1.54**
		Vilaboa	18	5.7	3.14	**2.05**	11	3.9	2.79	**2.16**
		Vilagarcía de Arousa	49	28.2	1.74	**1.58**	39	19.5	2.00	**1.72**
		Vilanova de Arousa	26	13.8	1.89	**1.73**	29	9.2	3.14	**2.40**

SMR = Standard Mortality Ratio. RR = Relative Risk. pp = posterior probability that RR>1.

**Figure 4:**
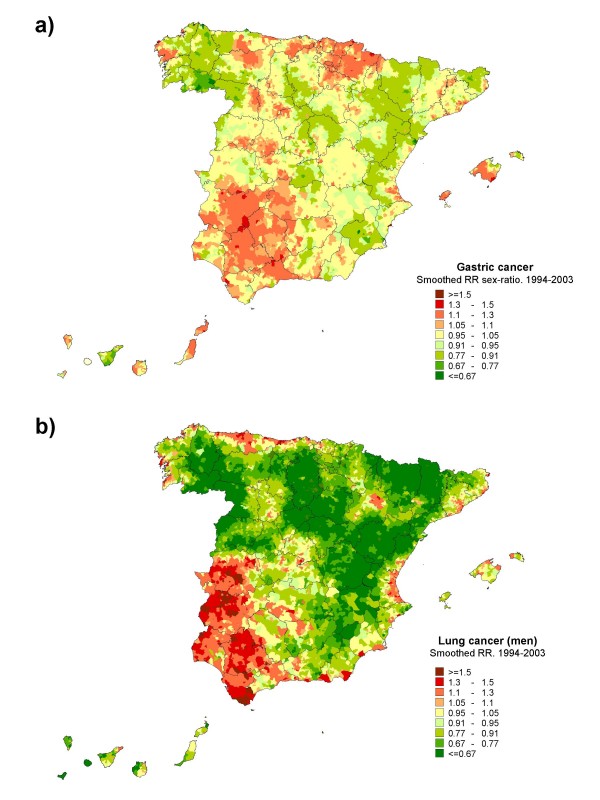
**Municipal distribution of: a) gastric cancer sex ratios obtained on the basis of smoothed RRs; b) municipal distribution of lung cancer mortality in men**.

Figure 4 depicts the sex ratios obtained on the basis of smoothed RRs, along with the distribution lung cancer mortality in men. It is noteworthy that the areas of highest risk of dying from lung cancer in men present the highest gastric cancer mortality sex ratios.

## Discussion

This study highlights the persistence of a marked geographic pattern in risk of gastric cancer mortality in Spain, which affects both sexes and which has not been observed for any other tumor. Special mention should be made of the high risk that extends across a broad swathe of the Northern Iberian Plateau (*Meseta*), covering the Autonomous Region of Castile & Leon and spreading northeast towards the Basque Country, Navarre and La Rioja, and west to Portugal and northern Extremadura. In addition, two further areas with excess risk were identified, namely, the Atlantic Coast of Galicia and the interior of Catalonia. This pattern's similarity in men and women and its maintenance over time suggest that distribution of stomach cancer here in Spain could be related to long-standing environmental exposures shared by both sexes, as gastric cancer is known to be the result of decades of interaction between chronic inflammation and exposure to carcinogens.

When it comes to interpreting the results, some factors must be taken into account. Firstly, mortality is not the best indicator for studying cancer distribution. However, mortality continues to be the only comprehensive source of cancer information in Spain. Though the geographic distribution of specific-cause mortality might be affected by differences in the quality of death certification between one part of the country and another, there are not too many arguments that could support possible inconsistencies and differences of criteria in the coding of death certificates. Gastric cancer is a well-certified cause of death in Spain, with detection and confirmation rates both exceeding 80% [21]. Another explanation for possible differences in cancer mortality among regions refers to variation in survival rates due to differences in the health care system across the country. Bearing in mind the characteristics of the Spanish National Health Care System, with universal accessibility, we would have no reason to suspect that there might be differential access to health care and diagnosis between regions.

The methodology used for the study of spatial disease patterns has traditionally relied on standardized mortality ratios (SMRs) [14]. For small geographic units, the use of SMRs introduces an extra source of variability, in the form of random variability, since sparsely populated areas with few or zero cases can generate extreme SMR values [14]. The Bayesian approach attempts to solve this problem, by using smoothing techniques that help identify the underlying geographic pattern. This approach is not entirely free of limitations, however, and there are authors who feel that Bayesian disease-mapping models are essentially conservative [20].

As previously mentioned, in Spain the gastric cancer mortality risk pattern is characterized by its singularity, the enormous similarity it displays across the sexes, and its persistence over time, inasmuch as its pattern has been known since the 1980s [22]. This pattern might partially be explained by geographic differences in the prevalence of *Helicobacter pylori *infection. This bacterium was classified as a carcinogen (Group 1) by the International Agency for Research on Cancer in 1994, due to its relationship with gastric cancer [23], and is currently viewed as the principal risk factor for noncardia gastric cancer [24]. Nonetheless the biologic pathways leading from *H. pylori *infection to gastric cancer are not yet well known. Despite the fact that over half the world population is infected, only a small percentage of infected subjects develop the cancer, and as *H. pylori *infection is equally prevalent in men and women, men have approximately twice the risk of gastric cancer. Prevalence of *H. pylori *infection displays important geographic differences worldwide, ranging from 40% or less in developed countries to 70% or more in developing countries [25]. According to published studies, Spain is situated in an intermediate position vis-à-vis industrialized and developing countries. Local studies have reported *H. pylori *infection prevalences from as low as 43% [26] to as high as 69% [27], but there are still wide areas of our country for which this information is lacking, not making possible to link data of gastric cancer mortality and prevalence of the infection.

Until the discovery of *Helicobacter pylori*, diet was the most important factor associated with gastric cancer. This is a very complex variable, that includes nutrient intake as well as exposure to contaminants. It is generally accepted that consumption of fruit and vegetables affords protection against the development of this tumor [28-30], whereas salt, and foods with higher levels of *N*-nitroso compounds are deemed to be risk factors [31-33]. In Spain, the foods that most contribute to exposure to nitrites and nitrosamines are meat products, cereals, vegetables and fruit in the case of the former, and processed meat, beer, cheese and broiled fish in the case of the latter [34]. Even so, there is no conclusive evidence as to the relationship between nitrogenated compounds and gastric cancer in humans. Moreover, the effect of such compounds could vary in accordance with intake of other substances, chemical or biologic contaminants and certain dietary components such as vitamins, *H. pylori *infection, and different patterns of genetic susceptibility [35].

The rising south-north and coast-inland gradient observed in the risk pattern, which is particularly marked in Castile & Leon and continues into Portugal [23,36] has been classically attributed to dietary habits, i.e., areas with regular consumption of cured, smoked and salted food, and low intake of fruit and fresh vegetables [37-40]. However, dietary patterns have changed, and Castile & Leon has become one of the Spanish regions with higher intake of vegetables and fruits.

An alternative explanation for the described pattern would be the existence of some environmental exposure linked to the geologic characteristics of this region. Areas with highest gastric cancer mortality in the Autonomous Region of Castile & Leon basically coincide with the Tertiary Duero River Basin, an area in which elevated levels of certain contaminants have been detected, both in soils and in underground waters, principal among which is arsenic [41-44]. Most of the towns affected by this problem, essentially farming communities, rely on underground water for crop irrigation [41,45]. Although the possible existence of contaminants associated with pesticide use is not ruled out, studies undertaken to date mainly link these high levels of arsenic to its presence in the rocky substrate and its subsequent seepage into underground water through natural geochemical mechanisms [41], which in turn suggests that exposure to this toxin among residents in such areas could go back many years.

The presence of arsenic in underground water in concentrations above WHO drinking-water guideline limits is a problem of enormous importance in many areas of the world. Curiously, many of the affected countries register the highest rates of gastric cancer worldwide, as is the case with China and Japan.

A possible role of arsenic in gastric carcinogenesis, whether directly or as a co-factor that facilitates the action of another mutagenic agent, seems to be a plausible hypothesis. It is a highly toxic compound that affects the gene repair pathways [46,47] and may, moreover, cause gastric irritation [48]. There is sufficient evidence to show that arsenic in drinking-water causes cancers of the urinary bladder, lung, and skin in humans, and might be related with liver or kidney neoplasms [49]. At present, however, there are few epidemiologic studies that have reported an association between exposure to arsenic and development of gastric cancer [50,51]. Notwithstanding, it is interesting to note that gastric cancer incidence and mortality decline in developed countries in the second half of the 20^th ^century, commonly attributed to improvements in food preservation and preparation, also coincides with a decrease in the consumption of water drawn from deep underground sources and the parallel rise in the number of persons supplied with potable water piped from surface sources.

There are other metals too that could also be present in the designated area, due fundamentally to industrial pollution, a major cause of water pollution. According to the data reported to the EPER in 2001, which lists industrial contaminant releases to air and water, Castile & Leon ranks second in Spain in terms of tons of chrome released directly into water, mainly into the tributaries of the Ebro and Duero Rivers [52]. In relation to chrome emissions, Burgos province -in Castile & Leon- ranks second at national level, only behind Tarragona, a coastal province which releases part of its waste into the Mediterranean Sea. Hexavalent chrome, a recognized carcinogen, raises the risk of gastric cancer in experimental studies [53]. Its genotoxic and mutagenic effect *in vitro *is boosted in the presence of certain dietary components [54]. Further data are still needed, however, to clarify the relationship between digestive tumors and exposure to this agent, which, in the general population, is essentially delivered by diet and drinking water.

Other toxins present in the Duero basin are nitrates [55]. Although these occur naturally in some groundwater, in most cases higher levels are thought to result from human activities. In Spain, though drinking water accounts for a small percentage of the total intake of these agents, gastric cancer mortality has been correlated with increasing exposure to nitrates in drinking water, not only in an area with high gastric cancer mortality rates [56] but also in a low risk region [57,58]. While similar results have been reported in Hungary [59], there are studies that do not support this hypothesis [60]. It should be noted that nitrate pollution also indicates low water quality and so might be accompanied by other pollutants. Moreover nitrate contamination is a problem common to many Spanish aquifers, and is therefore not specific to high gastric cancer risk areas.

The excess risk displayed by both sexes in the interior of Catalonia has been previously described, and attributed to the more rural, inland population's dietary habits -associated with stomach cancer- and to the absence of readily accessible health centers [61]. Nonetheless, the implication of other environmental exposures should not be ruled out. Local aquifers are heavily overexploited, and Catalonia is home to the river basin registering the greatest use of water for human consumption in Spain [62].

With respect to the excess gastric cancer risk observed on the Atlantic Coast of Galicia, it is worth noting that the towns with the highest risks countrywide in both sexes are all situated on the Morrazo Peninsula (Pontevedra province), a small geographic area with over 90,000 inhabitants. The sex ratio in this area is close to unity, something which suggests the possible implication of environmental risk factors. The economy of these towns is based on fishing, preparation of dried and salted fish, and shellfishing, mussel breeding in particular. Contaminants present in the estuaries, such as certain microorganisms, chemical pollutants (heavy metals, persistent organochlorinated pollutants, and polycyclic aromatic hydrocarbons) and marine toxins can indeed rise to high levels in shellfish and crustaceans. Such toxicants would reach the gastric mucosa by ingestion of local fish and seafood. One of these biotoxins is okadaic acid, which is present in mussels and involved in diarrhetic shellfish poisoning. This toxin has been shown to behave as a tumor promoter in mice and been proposed as a cause of digestive cancers in humans [63].

Finally, it should be stressed that the map depicting the male:female ratio of estimated risks at a municipal level suggests that tobacco may play an important role in men in those areas where this ratio is higher, given the similarity between its spatial distribution and the male risk pattern of dying from tobacco-related tumors, such as lung and bladder cancer [64]. In Spain, the prevalence of female smokers until 1960-70 was very low [65]; in fact, time trend in lung-cancer mortality rates in women did not reflect changes in smoking patterns until the 90s, when a increase in mortality among younger generations was detected, showing the early phase of the smoking-related lung-cancer epidemic among Spanish females [66]. Although the role of tobacco in this tumor's development has been subject of debate for many years, recent studies are furnishing evidence supporting the fact that smoking is an important risk factor for cardia gastric cancer [67,68]. Our results are in accord with the consistent relationship found by other authors between tobacco and male stomach cancer, and the lower evidence of this association for females [69].

To sum up, this paper suggests possible environmental hypotheses that might help to explain the persistence of the peculiar spatial gastric cancer mortality pattern over time, similar across the sexes. Some environmental contaminants, such as chrome, arsenic, nitrites or marine toxins delivered via diet and drinking water, could act as genotoxic agents or as irritants of the gastric mucosa. The possible modifying role of those environmental toxicants on the effect of the principal known risk factors, including *H. pylori *infection, diet, or smoking, could be an interesting topic that would be worth bearing in mind in future studies.

## Abbreviations

AC: adenocarcinomas; ICD-9: international classification of diseases, 9th revision; RR: relative risk; SMR: standardized mortality ratio; ASR: age-standardized rate.

## Competing interests

The authors declare that they have no competing interests.

## Authors' contributions

GLA, NA, MP, and BPG were all involved in designing the study. GLA, RR and EV performed the statistical analysis. NA wrote the first draft of the manuscript to which all authors subsequently contributed. All authors made contribution to statistical analyses and interpretation of results, and revised the manuscript for important intellectual content. All authors read and approved the final manuscript.

## Pre-publication history

The pre-publication history for this paper can be accessed here:

http://www.biomedcentral.com/1471-2407/9/316/prepub
